# Investigation of the molecular mechanisms of action of sophorolipids in MCF-7 cells: therapeutic potential against breast cancer

**DOI:** 10.1007/s13205-026-05096-0

**Published:** 2026-10-23

**Authors:** Luiz Henrique Santana Martins, Luan Vitor Alves de Lima, Matheus Felipe da Silva, Niumaique Gonçalves da Silva, Maria Claudia Terkelli de Assis, Isabella Cristina Oliveira Carvalho, Isaura Maria Fuzzinato, Beatriz Braga Silva, Dimitri Alves da Costa, Mattheus Pizane de Azevedo Silva, Maria Antonia Pedrine Colabone Celligoi, Mario Sérgio Mantovani

**Affiliations:** 1 Department of General Biology, State University of Londrina, Londrina, Paraná Brazil; 2 Department of Biochemistry and Biotechnology, State University of Londrina, Londrina, Paraná Brazil

**Keywords:** Antiproliferative, Breast adenocarcinoma, Biosurfactants, Cytotoxicity, Glycolipids

## Abstract

A mixture of lactonic and acidic sophorolipids exerted concentration-dependent cytotoxicity against human breast ductal adenocarcinoma (MCF-7) cells, with an IC₅₀ of 82.54 µg/mL as determined by resazurin assay. Phase-contrast microscopy revealed morphological alterations including cell swelling, loss of adhesion, and impaired membrane integrity. Flow cytometry analysis demonstrated cell cycle arrest in the S phase, mitochondrial membrane depolarization, and a predominance of necrosis-like cell death at higher concentrations. Transcriptional profiling by RT-qPCR identified overexpression of *CDKN1A*, *GADD45A*, *ERN1*, and *BBC3*, alongside repression of *ACTB* and *GAPDH*, indicating activation of DNA damage and stress-response pathways, endoplasmic reticulum stress, structural collapse, and metabolic dysfunction. Collectively, these findings demonstrate that sophorolipids induce mitochondrial and endoplasmic reticulum stress, culminating in homeostatic failure and cell death in MCF-7 cells, positioning these biosurfactants as promising candidates for the development of natural antiproliferative therapies.

**Supplementary Information:**

The online version contains supplementary material available at https://doi.org/10.1007/s13205-026-05096-0.

## Introduction

Breast cancer remains the most common malignant neoplasm among women worldwide, with an estimated 2.3 million new cases and 670,000 deaths reported in 2022 (Kim et al. 2025). This corresponds to approximately 25% of new diagnoses and 15% of cancer-related deaths in women. Despite advances in screening and treatment, breast cancer continues to be marked by high incidence and pronounced regional disparities. Higher incidence rates are observed in countries with a high Human Development Index (HDI), such as Australia, New Zealand, and Northern Europe, whereas mortality rates are higher in regions with a low HDI, reflecting inequalities in early diagnosis and access to treatment (Kim et al. 2025). Projections indicate that by 2050, the annual number of new cases may reach 3.2 million, while deaths could exceed 1.1 million, representing increases of 38% and 68%, respectively, with a greater impact in low- and middle-income countries (Kim et al. 2025).

In Brazil, breast cancer is also the most prevalent type of cancer among women, second only to non-melanoma skin cancer. Its incidence is higher in South and Southeast regions, which is associated with increased urbanization and improved access to health services (INCA 2023). Given this high prevalence and the persistence of inequalities in clinical outcomes, the development of new therapeutic approaches that are more effective, accessible, and safe remains essential to reduce mortality and improve patients’ quality of life.

In this context, sophorolipids, natural glycolipids produced as secondary metabolites by the non-pathogenic yeast *Starmerella bombicola*, have attracted increasing attention due to their pharmacological properties (Shu et al. 2021). These molecules exhibit promising characteristics, such as biodegradability, low environmental toxicity, and efficient production by fermentation, which make them attractive for both industrial and biomedical applications. Currently, sophorolipids are widely explored in cosmetic formulations, detergents, and textile products (Minucelli et al. 2017; Cho et al. 2022; Pal et al. 2023). Structurally, sophorolipids consist of a hydrophilic (sophorose) portion, composed of two glucose molecules linked by a β-1,2-glycosidic bond, and a hydrophobic part (a saturated or unsaturated fatty acid). These components are connected by a β-glycosidic bond (Ma et al. 2020). Depending on the presence or absence of lactonization between these segments, sophorolipids are classified as acidic (open chain) or lactonic (cyclic structure) forms, with further structural related to carbon chain length, degree of unsaturation, and acetylation (Dolman et al. 2017; Jezierska et al. 2018; Cho et al. 2022).

Sophorolipids display a broad spectrum of biological activities, supporting their potential for biomedical and pharmaceutical applications. They demonstrate antibacterial (Wang et al. 2023), antiviral, and antifungal effects, primarily through disruption of the microbial cell membrane and interaction with the lipid envelopes of viruses such as HIV, Influenza and Herpes, leading to cell lysis and leakage of intracellular content (Borsanyiova et al. 2016; Shu et al. 2021; Filipe et al. 2022; Dahmer et al. 2023; Bigotto et al. 2025). In oncological contexts, sophorolipids have demonstrated cytotoxic activity and the ability to induce apoptosis or necrosis, depending on their chemical structure, concentration, and cell model studied (Haque et al. 2021). Apoptosis is mainly mediated via the intrinsic mitochondrial pathway, involving modulation of Bcl-2 family proteins (↑Bax/↓Bcl-2), mitochondrial outer membrane permeabilization, cytochrome c release, and activation of the APAF-1/caspase-9/caspase-3 cascade (Wang et al. 2021; Singh et al. 2025). In addition, endoplasmic reticulum stress-mediated apoptosis, involving CHOP and caspase-12, has been reported in HeLa cells, possibly associated with increased intracellular calcium levels (Li et al. 2017). Sophorolipids also promote the generation of reactive oxygen species (ROS), activating pro-apoptotic pathways such as JNK and p38 MAPK and contributing to mitochondrial dysfunction (Miceli et al. 2022).

Recent studies have investigated the antiproliferative potential of sophorolipids, demonstrating their ability to interfere with cell cycle progression and induce cell death in various tumor cell lines. In vitro evidence shows activity against several carcinomas, including esophageal (Shao et al. 2012), cervical (Nawale et al. 2017), lung, and breast cancers (Ribeiro et al. 2015; Miceli et al. 2022; Roberge et al.^2023^). These effects are associated with cell cycle arrest in the G_0_/G_1_ or G_2_/M phases, as well as reduced cell migration and invasion, suggesting a potential antimetastatic role in different in vitro tumor models (Li et al. 2021; Marcelino et al. 2021). Moreover, sophorolipids exhibit relatively low toxicity in non-malignant cell lines, a characteristic that may contribute to cytotoxic specificity and a lower likelihood of adverse effects in vivo (Li et al. 2017; Diaz et al. 2018).

Although the antiproliferative effects of sophorolipids have been demonstrated in various in vitro cancer models, including breast cancer, the underlying cellular and molecular mechanisms are not yet fully elucidated. Given the high incidence and mortality of breast cancer, as well as the existing disparities in clincal outcomes, further investigation into novel therapeutic strategies is needed. In this context, the present study aims to investigate the mechanisms of action of sophorolipids in MCF-7 cells, a human breast adenocarcinoma cell line, in order to better understand the antiproliferative effects and assess their potential as a therapeutic alternative for the management of breast cancer.

## Methodology

### Cell line and chemical agents

The MCF-7 human breast carcinoma cell line was obtained from the Cell Bank of Rio de Janeiro. Cells were cultured in 25cm^2^ culture flasks containing Dulbecco’s Modified Eagle Medium (DMEM) supplemented with 10% fetal bovine serum (FBS; Gibco). Cultures were maintained at 37 °C in a humidified atmosphere with 5% CO_2_, and tested negative for mycoplasma contamination.

Cell viability was determined prior to all experiments using trypan blue exclusion assay with an automated Countess™ II cell counter. Only viability greater than 85% was accepted. For each experiment, cells were seeded in three independent biological replicates, each in triplicate, and allowed to stabilize for 24 h before treatment.

Sophorolipids used in this study were provided by the Laboratory of Biochemistry and Biotechnology (Silva et al. 2026), and were previously identified and characterized, with lactonic and acidic fractions accounting for 63.15% and 36.85%, respectively. Sophorolipids were solubilized in distilled water (pH 7.4) and diluted in culture medium at the time of treatments. For experimental assays, doxorubicin 8 µM (DXR, Doxolem, Zodiac) was used as a positive control.

### Cytotoxicity testing

Cytotoxicity was assessed using the resazurin assay, as described by O’Brien et al. (2000). Cells were seeded in 48-well plates at a density of 1 × 10^5^ cells/well in 200 µL of culture medium. After the stabilization period, cells were treated with sophorolipids at different concentrations (40–120 µg/mL) with a final volume of 300 µL per well and incubated for 24 h.

Following treatment, 50 µL of resazurin solution (60µM) was added to each well, and the plates were incubated at 37 °C for 4 h. Fluorescence was then measured using a Glomax^®^ spectrophotometer with excitation of 520 nm and emission between 580 and 640 nm. Cell viability was calculated using the following equation:

1 $$\:Percentage\:of\:viability\:=\:\left[\frac{\left(Treatment\:A\:-\:White\:A\right)}{(control\:A\:-\:White\:A)}\:\times\:100\right]$$

Equation 1 Calculation of relative cell viability, where A represents the fluorescence intensity value.

The half maximal inhibitory concentration (IC_50_), as well as IC_25_ and IC_12.5_ values and the correlation coefficient (R^2^) were determined using GraphPad Prism 9.0 (GraphPad Software, USA).

### Cell morphology analysis

For morphological analysis, cells were seeded in 24-well plates (1.75 × 10⁵ cells/well) and treated with sophorolipids at IC_50_, IC_25_, and IC_12.5_ concentrations for 24 h. Cells were then incubated for 30 min at 37 °C, protected from light, with the following staining solutions: (i) Hoechst 33,342 and propidium iodide (PI) for the assessment of nuclear integrity and plasma membrane compromise, and (ii) Hoechst 33,342 and rhodamine 123 (Rh123) for the evaluation of nuclear integrity and mitochondrial activity. Images were acquired using an EVOS FL AMAFD1000 inverted fluorescence microscope (Invitrogen, Thermo Fisher Scientific, USA) under both brightfield and fluorescence channels. This dual staining approach allowed simultaneous assessment of nuclear morphology, mitochondrial activity, and membrane integrity after treatment with sophorolipids.

### Apoptosis and cell cycle assay by flow cytometry

To evaluate whether sophorolipids induce cell death via apoptosis and/or necrosis, the Guava Nexin^®^ apoptosis kit (Merck/Millipore, 4500–0450) was used. This assay detects the externalization of phosphatidylserine through the interaction between phosphatidylserine and annexin V, allowing the quantification of the following cell populations: viable cells [Annexin V (−)/7-AAD (7-aminoactinomycin D) (−)], early apoptotic cells [Annexin V (+)/ 7-AAD (−)], late apoptotic cells [Annexin V (+)/ 7-AAD (+)] and dead cells [Annexin V (−)/7-AAD (+)].

For cell cycle analysis, DNA content was measured to determine the distribution of cells in each phase (G_1_, S, and G_2_/M). Cells were permeabilized with 100 µL of a citrate/Triton solution (0.1% sodium citrate/0.1% Triton X-100), and the DNA was stained with 5 µL of propidium iodide (50 µg/mL) (Lima et al. 2022).

Cells were seeded in 6-well plates at a density of 1 × 10⁶ cells/well and incubated for 24 h. After this period, cells were treated with sophorolipids at concentrations corresponding to IC_50,_ IC_25_, and IC_12.5_, along with control groups treated with DXR (8 µM) and vehicle (PBS), for 24 h. Following treatment, cells were washed with PBS, trypsinized (0.025%), and centrifuged at 900 × g for 5 min. The resulting cell pellet was resuspended in 200 µL of PBS. In both assays, the fluorescence intensity of each marker was determined using a flow cytometer (Guava EasyCyte), considering 5,000 events/sample.

### Mitochondrial membrane potential analysis (Δψm)

Rh123 was used to estimate the mitochondrial membrane potential (Δψm), based on its mitochondrial accumulation, with fluorescence emission proportional to membrane potential. In total, 1 × 10^6^ cells/well were seeded in 6-well plates and incubated for 24 h. Cells were then treated with sophorolipids at IC_50_ and IC_25_ concentrations, and positive control (DXR) for another 24 h. After treatment, 2 µL of rhodamine 123 solution (1 mg/mL) was added to each well, and the plates were incubated for 30 min, protected from light. Cells were, finally, collected and centrifuged at 900 × g for 5 min and resuspended in 100 µL of PBS. Fluorescence intensity was measured by flow cytometry (Guava EasyCyte) using a red filter, based on 5,000 events per sample, to determine the mean fluorescence of cells (Biazi et al. 2017).

### Gene expression analysis by RT-qPCR

Reverse transcription quantitative PCR (RT-qPCR) was conducted to evaluate relative mRNA expression of genes related to molecular pathways of cell death, oxidative stress, endoplasmic reticulum stress, DNA damage, cell cycle regulation, and autophagy in MCF-7 cells treated with IC_50_ of sophorolipids. The *RPLP0* gene was used as the reference gene.

Total RNA was extracted using the RNeasy^®^ Mini Kit (Qiagen), according to the manufacturer’s instructions. RNA concentration and purity were assessed using a BioDrop µLite spectrophotometer based on the absorbance ratio (A260/280 nm), and only samples with values between 1.9 and 2.0 were used. cDNA synthesis was performed in a T100™ Thermal Cycler (Bio-Rad). Each reaction contained 500 ng of total RNA diluted in 12.4 µL of DEPC-treated water, 1 µL of oligo dT (20 pmol/µL; Invitrogen, cat. no. 18418012), and 2 µL of dNTPs (10 mM; Invitrogen, cat. no. 10297018) and was kept at 60 °C for 15 min. Subsequently, 0.1 µL of RNase OUT (4 units, Invitrogen, cat. no. 10777019), 4 µL of 5× first-strand buffer (Invitrogen, cat. no. Y02321), and 0.5 µL of M-MLV-RT (Invitrogen, cat. no. 28025–013) were added. The samples were incubated at 37 °C for 2 min, 50 °C for 45 min, and 70 °C for 15 min.

qPCR was carried out using a LightCycler^®^ Nano (Roche) thermal cycler. The amplification protocol consisted of an initial step of denaturation at 95 °C for 5 min, followed by 40 cycles of 95 °C for 20 s, 60 °C for 30 s, and 72 °C for 30 s. Each RT-qPCR reaction contained 12 µL of final volume, including 5 µL of diluted cDNA (1:10), 0.5 µL of each primer (forward and reverse), and 6 µL of QuantiNova SYBR Green PCR Mix (Qiagen, cat. no. 208052). At the end of the amplification, the melting curve analysis was performed from 50 °C to 90 °C, with increments of 0.5 °C every 20 s (Lima et al. 2022).

### Statistical analysis

The data obtained were submitted to the Shapiro-Wilk test for normality verification. Parametric data were analyzed by one-way analysis of variance (one-way ANOVA), followed by Dunnett’s post-hoc test for multiple comparisons with GraphPad Prism^®^ 9.0 software (GraphPad Software, Inc.). Nonparametric data were submitted to the Mann-Whitney test. Gene expression data were assessed using the 2^−∆∆Ct^ method, considering fold-change values ≤ 0.5 and ≥ 2 as biologically significant. Results are presented as mean ± standard deviation (SD), with a significance level of 5% (*p* < 0.05).

## Results

### Cytotoxicity

**Fig. 1:**
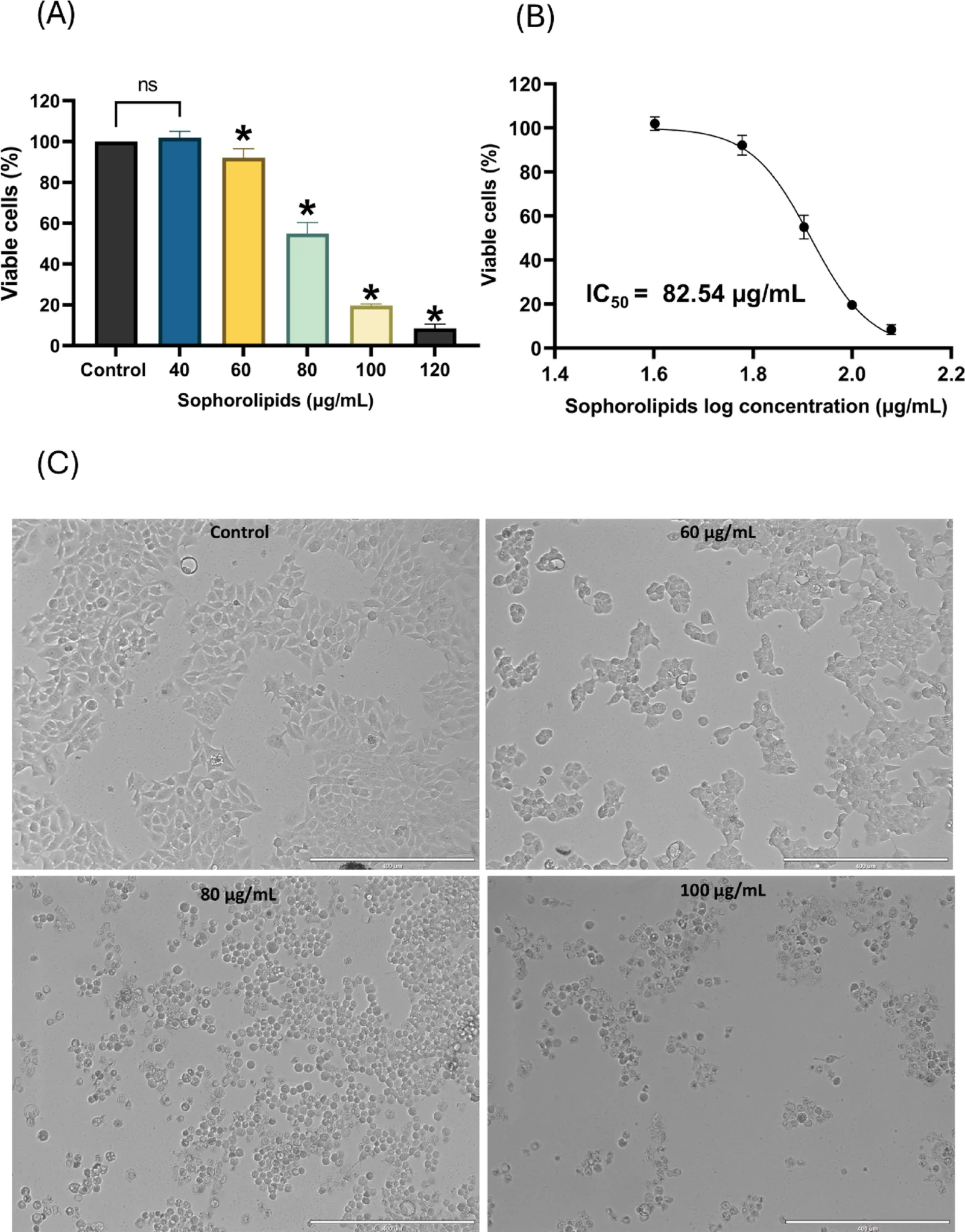
Cytotoxic effect of sophorolipids on the mammary adenocarcinoma cell line (MCF-7). **A** Cell viability determined by the Resazurin assay after 24 h of exposure to various concentrations of sophorolipids (40–120 µg/mL), Doxorubicin was used as a positive control (see Supplementary Data). **B** Non-linear regression curve (dose-response) used to calculate the half-maximal inhibitory concentration IC_50_ and the IC_25_ and IC_12.5_ values. **C** Representative phase-contrast microscopy photomicrographs of MCF-7 cells after 24 h of treatment. The images highlight morphological alterations, including cell swelling, loss of morphology, reduced confluency, and cell detachment associated with cell death at higher concentrations. Scale bar 400 μm. Data are expressed as the mean ± standard deviation of three independent experimental replicates. Asterisks (*) indicate statistically significant differences compared to the control (*p* < 0.05) as determined by one-way Analysis of Variance (ANOVA) followed by Dunnett’s post-hoc test

After 24 h of treatment with sophorolipids, a reduction in cell viability was observed (Fig. 1) at concentrations of 60 µg/mL (92.10 ± 4.489%), 80 µg/mL (54.96 ± 5.351%), 100 µg/mL (0.8367), and 120 µg/mL (8.401 ± 2.168%). This effect occurred in a dose-dependent manner with a significant coefficient *R* = − 0.9809. The IC_50_, IC_25_, and IC_12.5_ values were calculated at 82.54 µg/mL, 70.90 µg/mL, and 63.05 µg/mL, respectively, and were used for subsequent tests.

### Cell morphology analysis

**Fig. 2:**
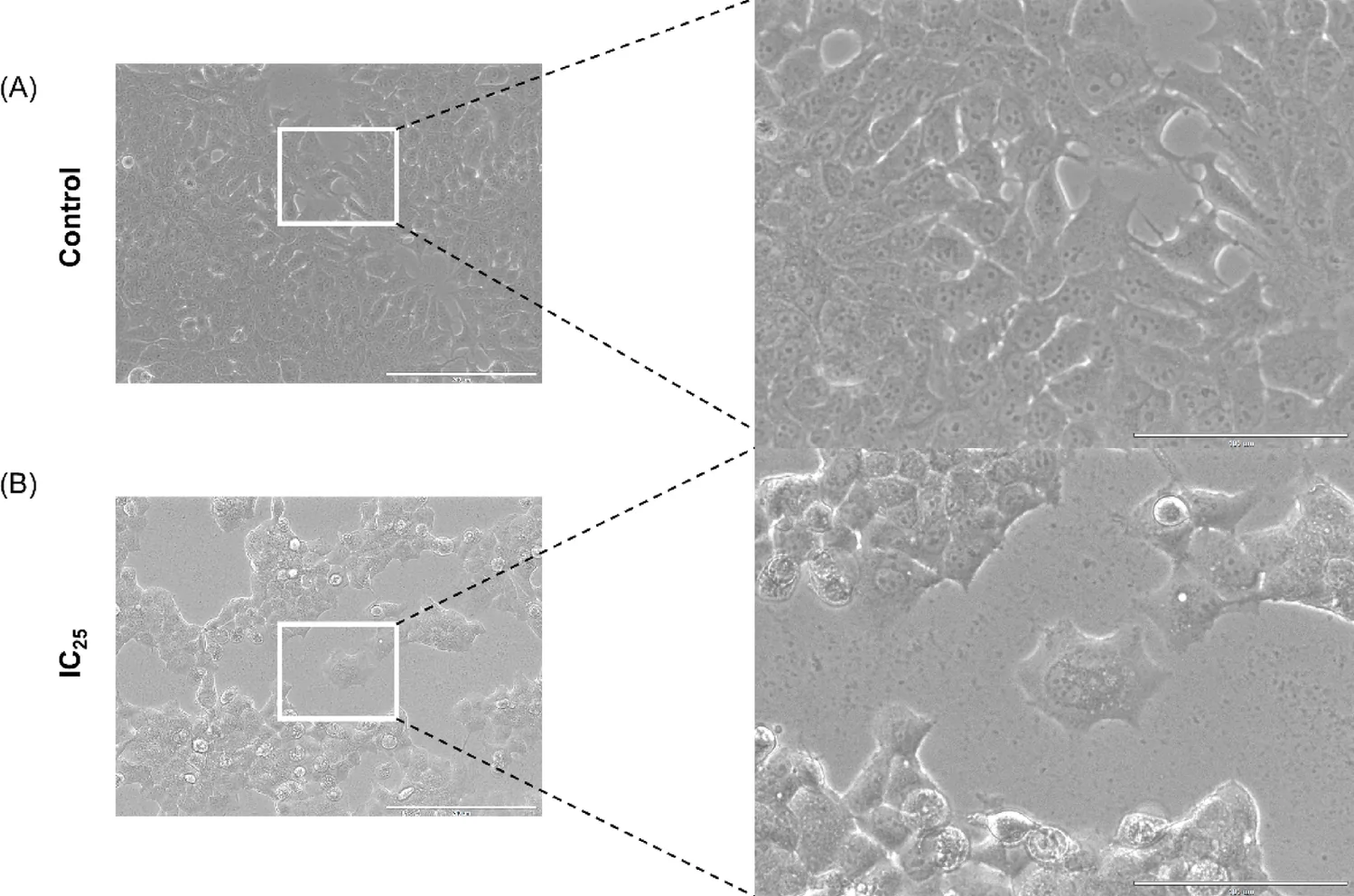
Morphological analysis of MCF-7 cells via phase-contrast microscopy. **A** Untreated control group after 24 h of incubation, displaying typical epithelial-like morphology, high cell confluency, and intact cell-to-cell adhesion. **B** MCF-7 cells treated with the IC_25_ of sophorolipids (70.90 µg/mL) for 24 h. Notable morphological alterations include significant cell swelling, loss of characteristic polygonal shape, and a visible reduction in cell density compared to the control

After 24 h of treatment of MCF-7 cells with sophorolipids at IC_50_, IC_25_ and IC_12.5_ concentrations, brightfield analysis revealed morphological alterations, including cell swelling, reduced confluency, and increased detachment at higher concentrations, consistent with cell death (Fig. 2).

**Fig. 3:**
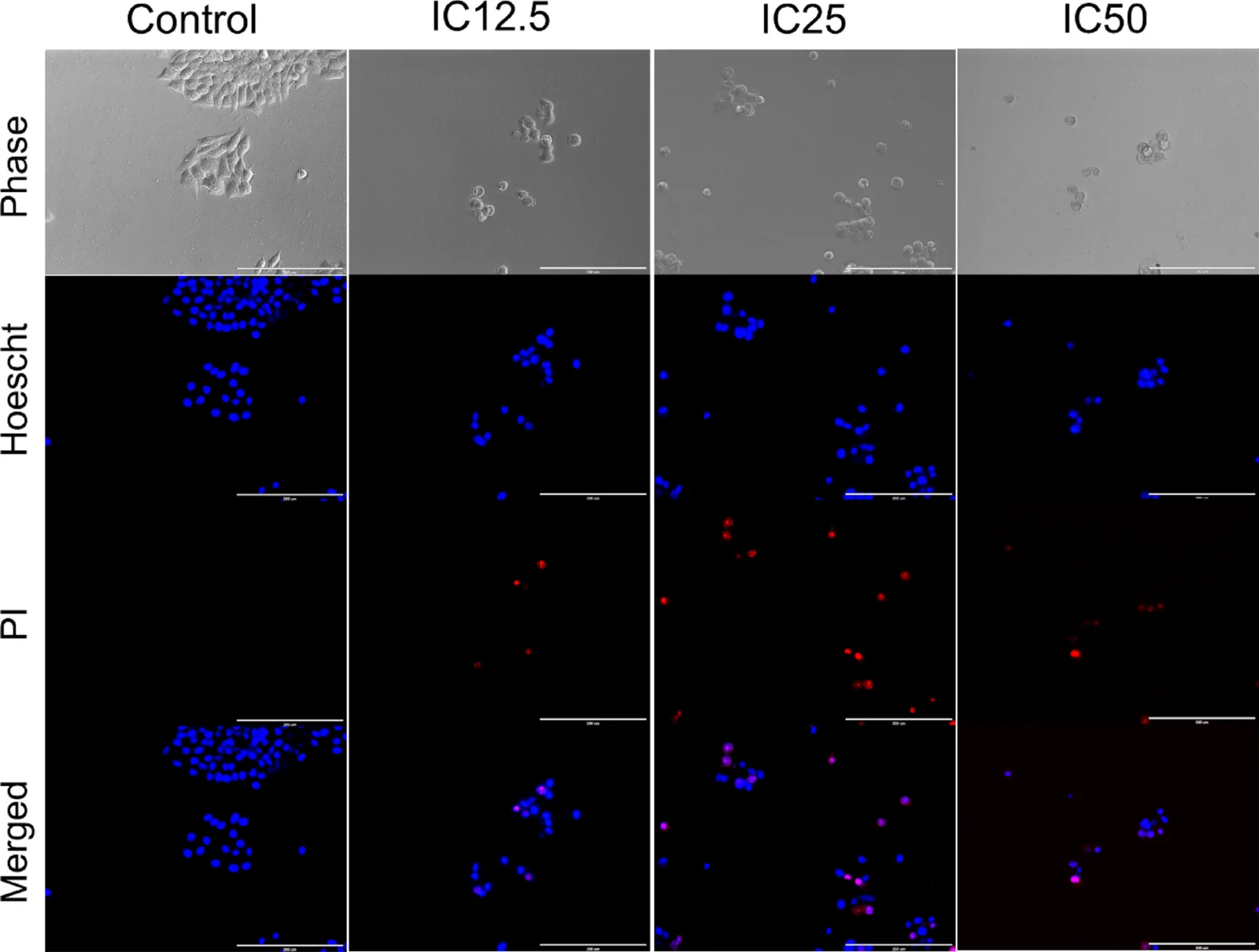
Fluorescence microscopy analysis of membrane integrity and nuclear morphology in MCF-7 cells. Representative images of MCF-7 cells after 24 h of treatment with sophorolipids at IC_25_ and IC_50_ concentrations. Blue fluorescence (Hoechst): labels cell nuclei, showing no significant chromatin condensation or nuclear fragmentation across groups. Red fluorescence (Propidium Iodide - PI): indicates compromised plasma membrane integrity. Untreated control showing intact membranes (PI-negative) and high confluency. IC_25_ treatment showing PI-positive cells, indicating initial membrane damage. IC_50_ treatment showing a marked reduction in cell density. Note that the lower intensity of PI signal at IC_50_ compared to IC_25_ is attributed to extensive cell detachment and loss of confluency, resulting in fewer cells remaining in the analytical field, rather than reduced cytotoxicity

Fluorescent (i) staining with Hoechst 33,342 and PI did not show evident changes in nuclear morphology between control and treated groups. However, a decrease in cell density was observed in groups exposed to the compound. PI staining was positive in treated groups, indicating loss of plasma membrane integrity, which is consistent with the observed morphological changes such as loss of adhesion and cell viability (Fig. 3).

**Fig. 4:**
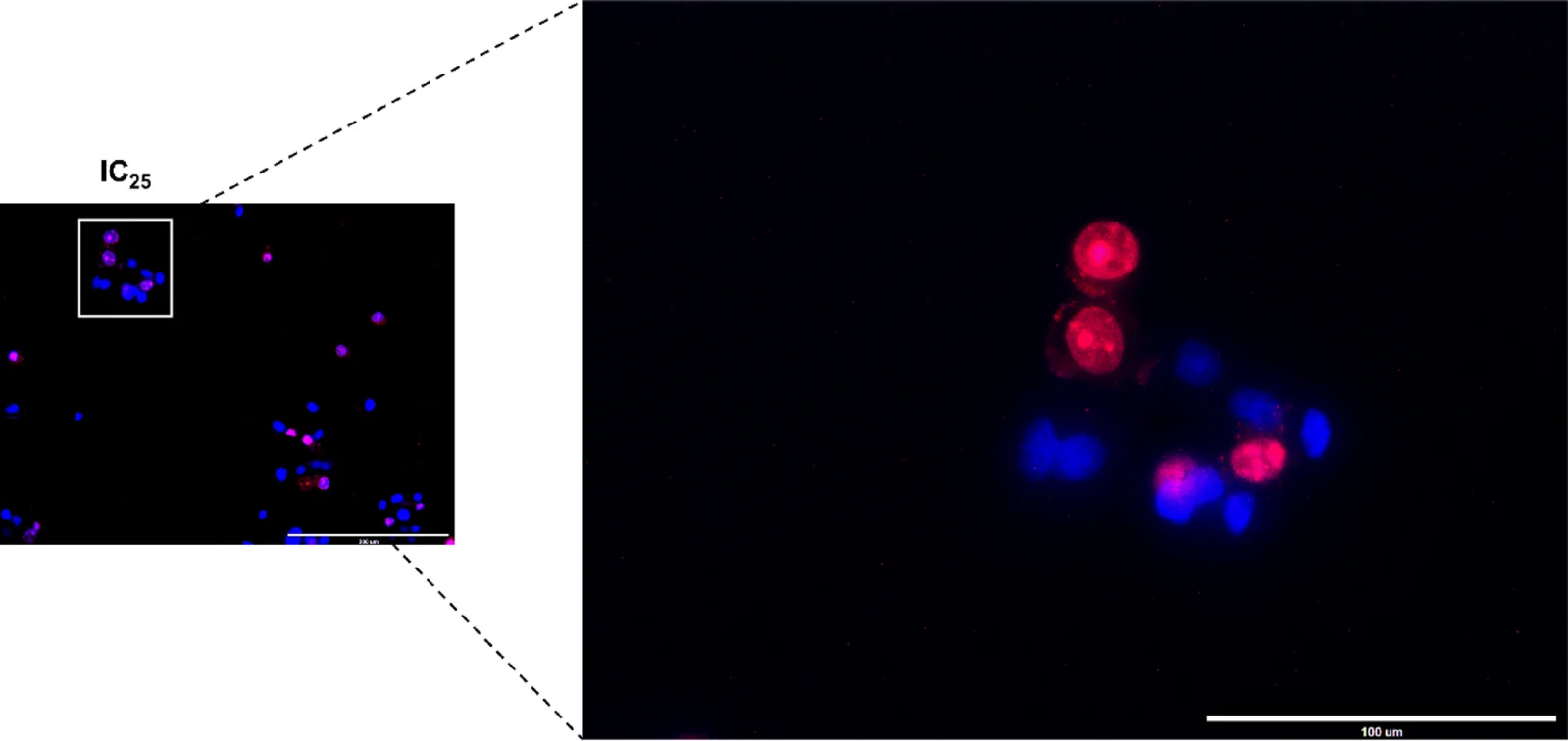
High-magnification analysis of nuclear morphology and membrane integrity in MCF-7 cells treated with the IC_25_ of sophorolipids (24 h). Representative merged fluorescence images (Hoechst/Propidium Iodide) demonstrating the intracellular localization of the markers after 24 h of treatment. The blue signal highlights nuclear structure, while the red signal (PI) confirms the loss of plasma membrane integrity. The high-magnification field reveals detailed cellular features, supporting the evidence of membrane disruption observed in the overall analysis

A lower PI signal at the IC₅₀ concentration was observed, likely due to the marked reduction in cell confluency and adhesion following treatment. This reduction may have decreased the number of cells present in the field of analysis, thereby affecting fluorescence intensity without necessarily indicating reduced membrane damage in the remaining cells. (Fig. 4).

**Fig. 5:**
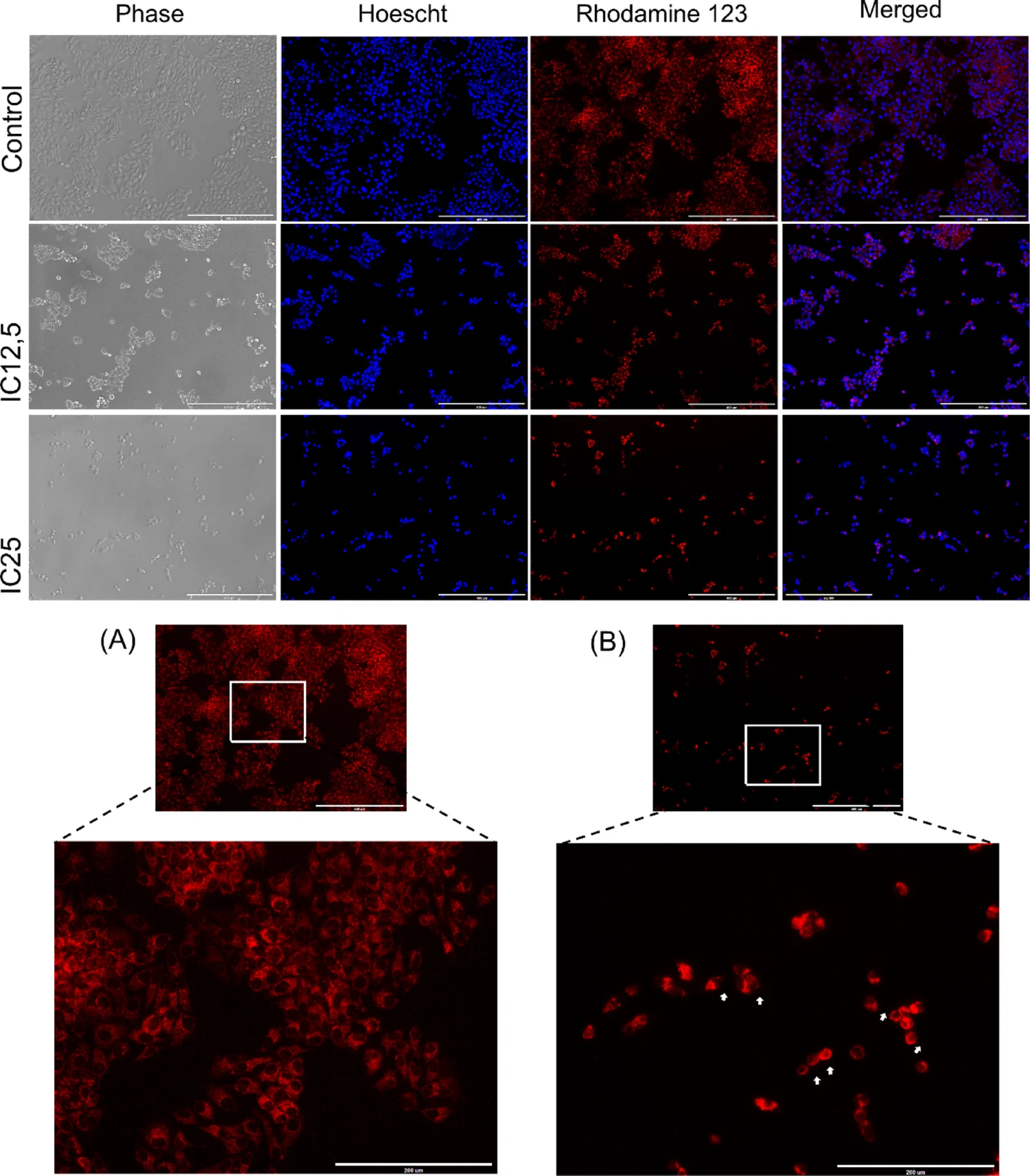
Assessment of nuclear morphology and mitochondrial distribution in sophorolipid-treated MCF-7 cells. The upper panel presents representative photomicrographs after 24 h of treatment at IC_12.5_ and IC_25_ concentrations, compared to the untreated control. Columns display phase-contrast, Hoechst nuclear staining (blue), Rhodamine 123 mitochondrial staining (red), and merged images. Nuclear morphology remained without evident alterations (e.g., no condensation) across all groups. The lower panel highlights higher-magnification areas (indicated by white boxes) of the Rhodamine 123 staining for the Control (**A**) and the IC_25_ group (**B**). In (**A**), mitochondria are observed to be uniformly dispersed throughout the cytoplasm of control cells. In contrast, images in (**B**) reveal alterations in the labeling pattern, with white arrows indicating the redistribution and accumulation of mitochondria in a predominantly perinuclear region, suggesting cellular stress mechanisms

On fluorescent staining (ii) with Hoechst 33,342 and Rh123 after 24 h of treatment with sophorolipids at concentrations of IC_12.5_, IC_25_, and IC_50_, the nuclei did not present relevant changes in relation to the control, maintaining the pattern previously observed. However, mitochondrial staining with Rh123 revealed changes in organelle distribution. In control cells, mitochondria were uniformly dispersed throughout the cytoplasm, whereas treated cells exhibited a more perinuclear accumulation pattern. This finding suggests that sophorolipids may influence mitochondrial organization and dynamics, possibly in response to cellular stress (Fig. 5).

### Sophorolipids induced S-cycle arrest

**Fig. 6:**
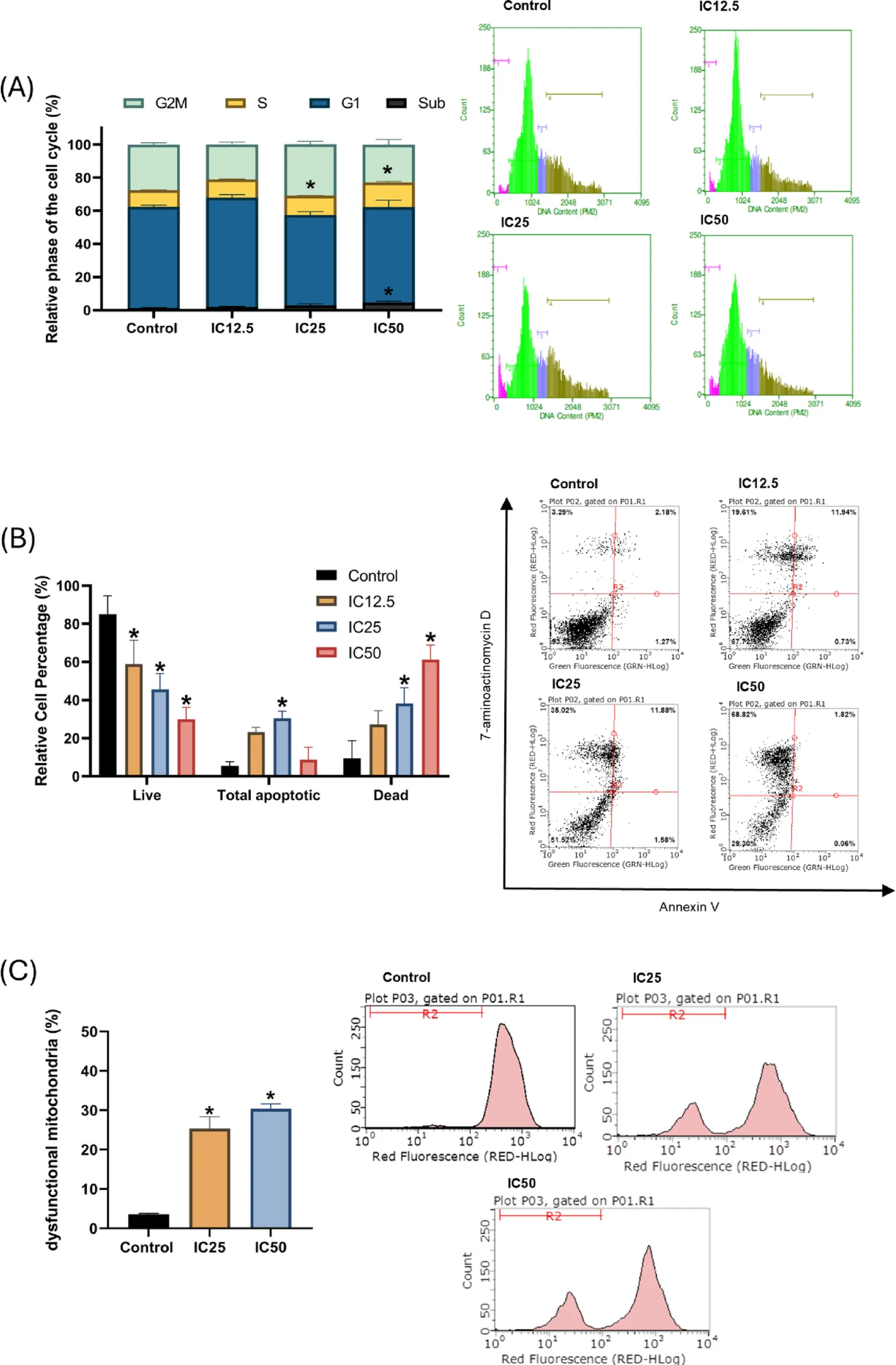
Flow cytometry analysis of cell cycle progression, cell death, and mitochondrial membrane potential in MCF-7 cells. Cells were evaluated after 24 h of exposure to sophorolipids at IC_12.5_, IC_25_, and IC_50_ concentrations. **A** Percentage distribution of cell cycle phases determined by DNA content quantification using propidium iodide (PI). The treatment induced a concentration-dependent accumulation of cells in the S phase (cell cycle arrest). **B** Proportion of viable, apoptotic, and necrosis-like dead cells obtained by Annexin V and 7-AAD dual labeling. A significant reduction in cell viability and a predominance of necrosis-like cell death are observed at higher concentrations. **C** Assessment of mitochondrial membrane potential *(*$\:\varDelta\:\varPsi\:m)\:$using Rhodamine 123. The increase in the percentage of cells with low fluorescence (R2) indicates mitochondrial membrane depolarization in response to treatments. Values are expressed as the mean ± standard deviation of three independent experimental replicates. Asterisks (*) indicate a statistically significant difference compared to the untreated control (*p* < 0.05), according to one-way Analysis of Variance (ANOVA) followed by Dunnett’s post-hoc test

After 24 h of treatment, sophorolipids altered the cell cycle distribution of MCF-7 cells. Compared to the control group (G_1_: 61.03 ± 1.72%; S: 10.00 ± 0.46%; G_2_/M: 27.53 ± 1.86%), cells treated with IC_50_ showed a significant increase in the S phase fraction (14.87 ± 1.12%) accompanied by a reduction in the G_2_/M (22.93 ± 5.10%). At the IC_25_ concentration, an increase in the S phase (11.63 ± 0.38%) was also observed, while the IC_12.5_ group showed a distribution similar to the control. These findings indicate that sophorolipids interfere with cell cycle progression, leading to accumulation of cells in the S phase, particularly at higher concentrations (Fig. 6a).

### Effect of sophorolipids on mitochondrial membrane potential ($\:\varDelta\:\varPsi\:m)$

After 24 h of exposure to sophorolipids at IC₅₀ and IC₂₅ concentrations, MCF-7 cells showed an increased percentage of cells with low Rh123 fluorescence, indicating mitochondrial membrane depolarization compared to the control group. The mean percentages of cells with reduced Δψm were 3.60% in the control, 25.33% in IC_25_ and 30.36% in the IC_50_ treatment; all while the positive control with DXR showed a similar depolarization profile. These results indicate that sophorolipids impair mitochondrial function in a concentration-dependent manner, possibly associated with mitochondrial stress processes and necrosis-like cell death (Fig. 6c).

### Influence of sophorolipids on cell death

Flow cytometry analysis allowed the evaluation of cell death induced by sophorolipids based on phosphatidylserine externalization and plasma membrane integrity (Fig. 6b). In the control, most cells remained viable (85.07%), with low proportions of necrosis-like death (4.05%) and apoptosis (5.52%). In contrast, treated groups showed a concentration-dependent increase in cell death. At the IC_12.5_ concentration, an increase in the population in necrosis-like death (27.30%) and apoptosis (23.27%) was observed, resulting in a significant reduction of viable cells (52.96%). At IC_25_, necrosis-like death predominated (40.32%), while apoptosis was less pronounced (11.68%), and cell viability decreased to 42.59%. At IC_50_, necrosis was the predominant form of cell death (59.31%), followed by apoptosis (14.55%), with a marked reduction in viable cells. Statistical analysis revealed significant differences for necrosis-like death at IC_25_ and IC_50_ groups, in apoptotic cells at IC_12.5_, and in viable cells at all tested concentrations. These findings indicate that sophorolipids reduce cell viability in a concentration-dependent manner, predominantly inducing necrosis-like death.

### Effect of sophorolipids on gene expression

**Fig. 7:**
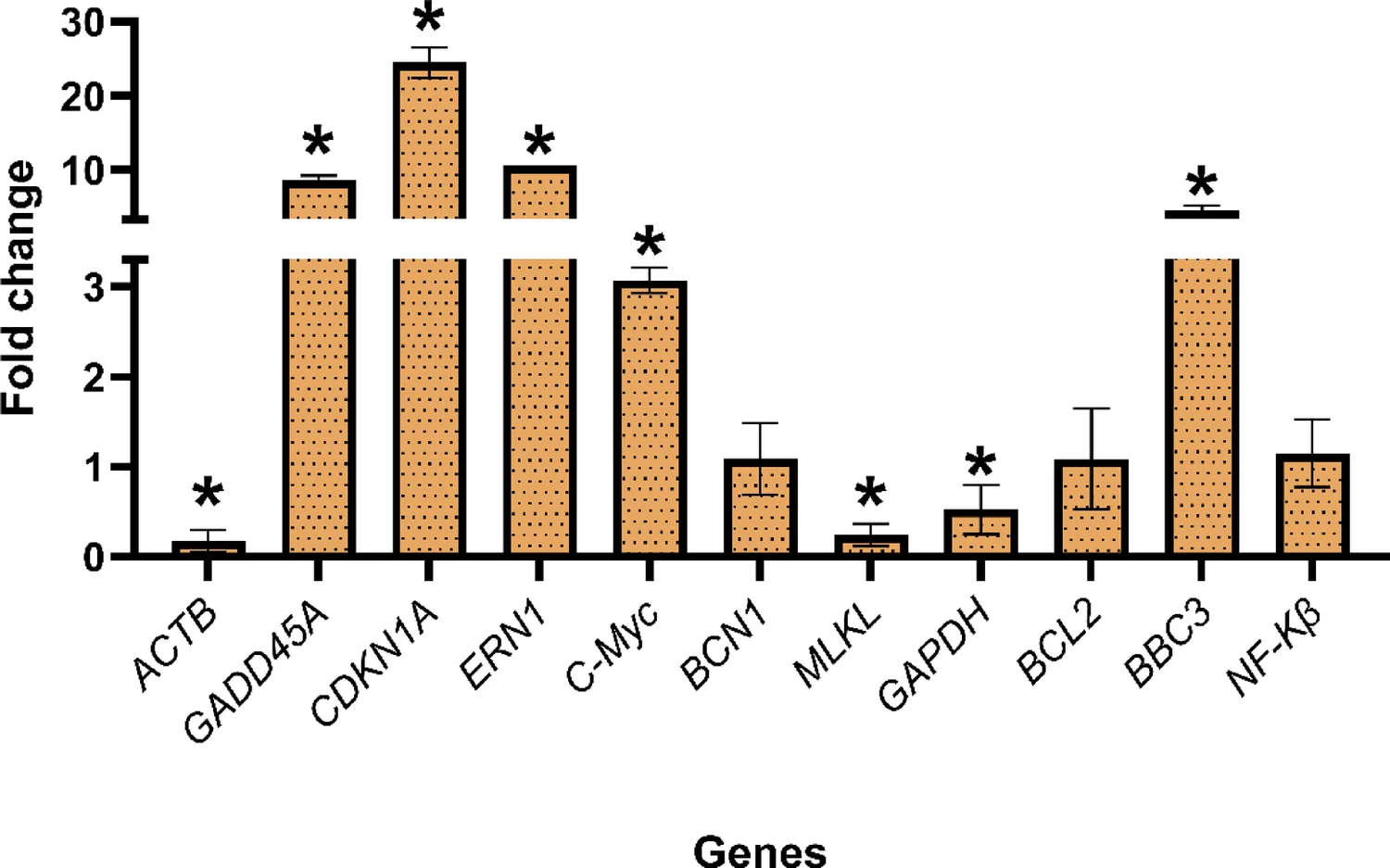
Relative expression of mRNAs in MCF-7 cells treated with sophorolipids at the concentration corresponding to IC₅₀, compared to the control group. The values are presented as mean ± standard deviation of three independent experiments. The analysis was performed considering significant differences when *fold change* ≤ 0.5 (repression) or ≥ 2 (overexpression), with a significance level of 5% (*p* < 0.05). The symbol (*) indicates a statistically significant difference in relation to the control

The expression levels of the analyzed genes were normalized using RPLP0 as the reference gene. *RPLP0* encodes a ribosomal protein and has been widely used due to its stable expression profile. In the present study, *RPLP0* exhibited consistent Qc (quantification cycle) values across control (17.057, 17.393, 16.770) and treated groups (17.604, 17.441, 17.441), supporting its suitability for normalization. Regarding relative gene expression, analysis revealed an increase in the expression of mRNAs of *CDKN1A* (Log_2_FC = 4.49), *GADD45A* (Log_2_FC = 3.11), *ERN1* (Log_2_FC = 3.40), *BBC3* (Log_2_FC = 2.54) and *C-MYC* (Log_2_FC = 1.61) genes following 24 h of treatment with sophorolipids at IC50 in MCF-7 cells. In contrast, expression levels of *MLKL* (Log_2_FC = -2.16), *GAPDH* (Log_2_FC = -1.45) and *ACTB* (Log_2_FC = -2.88) were reduced. The mRNAs of the *BCL2*, *BECN1*, and *NF-κB* genes remained stable, i.e., they did not show alterations (Fig. 7).

## Discussion

The evaluation of the effects of sophorolipids on the MCF-7 human breast carcinoma cell line is part of the growing interest in the antiproliferative potential of these microbial biosurfactants (Miceli et al. 2022). Produced by non-pathogenic yeasts such as *Starmerella bombicola*, sophorolipids stand out for their high fermentative efficiency and economic viability, making them promising candidates for low-cost therapeutic agents (Singh et al. 2025). In the present study, the sophorolipid mixture composed of 63.15% lactonic and 36.85% acidic fractions, showed an IC₅₀ value of 82.54 µg/mL in MCF-7 cells, confirming its cytotoxic potential, as previously reported in other tumor cell lines (Akiyode et al. 2016).

Mitochondrial functional analysis revealed a significant depolarization in ΔΨm, evidenced by an increased proportion of cells with low Rh123 fluorescence at IC_25_ and IC_50_ concentrations. These findings support the hypothesis of mitochondrial pathway involvement in cellular stress induced by sophorolipids, a mechanism also described in previous studies (Miceli et al. 2022; Nawale et al. 2017). However, the concomitant upregulation of the *ERN1* gene mRNA, a classic marker of endoplasmic reticulum (ER) stress, suggests that both organelles participate in the treatment response in an integrated manner. While Naweale et al. (2017) observed mitochondrial apoptosis mediated by increased intracellular Ca²⁺ in HeLa cells, Li et al. (2017) demonstrated that C18:1 diacetylated sophorolipids predominantly activate ER stress, regardless of mitochondrial involvement. In the present study, the coexistence of mitochondrial depolarization and *ERN1* mRNA overexpression suggests that sophorolipids simultaneously trigger both stress pathways, resulting in a severe homeostatic imbalance that culminates in cell death.

In addition, treatment of MCF-7 cells with sophorolipid mixture induced cell cycle arrest in the S phase at IC_50_ and IC_25_ concentrations. In particular, at IC_50_, a significant increase in the proportion of cells in the S phase was observed, accompanied by a reduction in the G_2_/M phase. At the molecular level, these effects were favored by the upregulation of *CDKN1A* and *GADD45A* mRNA expression. *CDKN1A* gene, a cyclin-dependent kinase inhibitor (CKI), plays a key role in cell cycle regulation, consequently promoting cell cycle disruption, while the increase in *GADD45A* expression may be linked to cellular responses to genomic damage. This may lead to checkpoint activation and S-phase arrest. The phase-specific arrest observed in MCF-7 cells differs from previous reports in other cancer cell lines treated with sophorolipids, highlighting the influence of both cell type and sophorolipids structure on the mechanism of action (Miceli et al. 2022).

Analysis of cell death revealed a predominance of necrosis-like death, reaching 59.31% of the cells treated at IC₅₀ concentration. This finding, together with the positive marking for PI staining, and the observed morphological changes, including cell swelling and detachment, indicates loss of plasma membrane integrity. Although apoptosis is frequently reported in cells treated with sophorolipids, such as in HepG2 cells where lactonic fraction activates the caspase-3 pathway (Wang et al. 2021), necrosis has also been described as a predominant response in certain contexts, likely related to the amphiphilic properties of these molecules and their interaction with cell membranes (Marcelino et al. 2021; Miceli et al. 2022). Thus, the present findings and available studies suggest that the type of death induced by sophorolipids is strongly related to both their chemical composition and the target cell type, with membrane disruption playing a central role in MCF-7 cells (Callaghan et al. 2022).

Transcriptional analysis further demonstrated significant modulations of genes associated with stress and cell death pathways. The upregulation of *ERN1* mRNAs, which encodes the IRE1α stress protein, indicates activation of the ER stress pathway, consistent with previous findings in sophorolipids-treated HeLa cells (Li et al. 2017). However, while ER stress in HeLa cells was associated with apoptosis, in MCF-7 cells it appears to be linked to necrosis-like death, possibly reflecting a more severe stress response.

In addition, the increased expression of *BBC3*, a pro-apoptotic gene of the Bcl-2 family whose product promotes mitochondrial membrane permeabilization, is consistent with the ΔΨm loss observed in this study and reinforces the involvement of the mitochondrial pathway in the cellular response (Chipuk et al. 2009). However, the stable expression of *BCL2* mRNA, an anti-apoptotic gene typically repressed during apoptosis, indicates that the intrinsic apoptotic pathway was engaged but not fully executed. This discordance may be explained by the well-established caspase-3 deficiency of MCF-7 cells, resulting from a 47-base-pair deletion in exon 3 of the *CASP3* gene (Tian 2023). As caspase-3 is required for nuclear fragmentation and other classical apoptotic morphological features, its absence would allow BBC3-mediated mitochondrial permeabilization to proceed without completion of a typical apoptotic phenotype, consistent with the absence of nuclear condensation detected by Hoechst staining (Nagata 2000; Chipuk et al. 2009). Accordingly, despite the strong activation of stress and cell cycle arrest genes such as *CDKN1A*, *GADD45A*, and *ERN1*, the predominance of necrosis-like death indicates that the resulting cell death was driven primarily by physical membrane damage associated with the amphiphilic nature of the sophorolipids, rather than by completion of the mitochondrial apoptotic pathway. Furthermore, considering that *MLKL* is the terminal effector of necroptosis and that its activation typically depends on RIP1/RIP3-mediated phosphorylation rather than on transcriptional induction (Gong et al. 2019), the downregulation of *MLKL* mRNA argues against necroptosis as the operative regulated cell death pathway, rather than constituting direct evidence of physical membrane damage. Taken together with the morphological and PI staining findings described above, this supports a scenario in which membrane disruption occurs largely independently of the classical necroptotic machinery.

In parallel, the repression of *ACTB* and *GAPDH* mRNAs indicate structural disorganization and metabolic collapse, respectively. Reduced *ACTB* expression, which encodes β-actin, is particularly relevant, as it points to the disruption of the cytoskeleton, a mechanism by which sophorolipids inhibit cell migration and adhesion. Previous studies have shown that sophorolipids, especially lactonic forms, disrupt actin filaments in tumor cell lines such as A549, MDA-MB-231, and B16F10 (Haque et al. 2021), leading to loss of cell anchorage and roundness, phenomena also observed in the study. PI positivity and *ACTB* mRNA reduction reinforce the link between structural damage and necrosis-like cell death, indicating that cell membrane rupture, accompanied by cytoskeleton disruption, is a major determinant of cell death in MCF-7 cells (Callaghan et al. 2022). In contrast, stable *NF-κB* mRNA expression indicates that pro-survival and inflammatory signaling pathways were not significantly activated, while repression of *ACTB* and *GAPDH* mRNAs, genes related to structural maintenance and energy metabolism, suggests a generalized metabolic breakdown. On the other hand, the moderate increase in *C-MYC* mRNA expression may reflect an initial proliferative or stress response, which may later be suppressed by cell cycle blockade mediated by p21, the protein product of the *CDKN1A* gene.

Overall, the results demonstrate that sophorolipids trigger multifactorial cellular stress, characterized by the simultaneous activation of mitochondrial pathways (*BBC3*) and ER (*ERN1*) pathways, accompanied by cell cycle arrest (*CDKN1A*, *GADD45A*) and structural and metabolic disruption (*ACTB*, *GAPDH*). This set of responses indicates a generalized dysfunction of cellular homeostasis, in which multiple control systems are interdependently affected. Although evidence of activation of apoptotic pathways (*BBC3* mRNA increase) was detected, the lack of *BCL2* and *MLKL* repression, suggests that cytotoxicity induced by sophorolipids is primarily driven be membrane damage and exacerbated cellular stress, rather than the coordinated activation of classical programmed cell death pathways.

Although this study provides evidence of the potential of sophorolipids, several limitations should be considered. The experiments were conducted exclusively using the MCF-7 cell line in vitro model characterized by an inherent caspase-3 deficiency, which impairs the execution of the classical apoptotic pathway and may explain the observed predominance of necrosis-like cell death. Furthermore, the assays were performed using a mixture of different sophorolipids isoforms (lactonic and acidic), making it impossible to distinguish the individual effects of each structural form. Future studies should address these limitations by evaluating isolated sophorolipid fractions individually, extending the analysis to complex models that better resemble in vivo conditions, including non-malignant controls, while incorporating protein-level validation of the proposed pathways, and ultimately assessing the efficacy and safety of sophorolipids in vivo.

## Conclusion

Sophorolipids produced by *Starmerella bombicola* exhibited significant cytotoxic activity against MCF-7 breast carcinoma cells, promoting mitochondrial membrane depolarization, S-phase cell cycle arrest, and predominantly necrotic-like cell death mechanism, accompanied by transcriptional modulation of cellular stress response (overexpression of *CDKN1A*,* GADD45A*,* ERN1*, and *BBC3*; repression of *ACTB* and *GAPDH).* These findings suggest that the cytotoxic effects of sophorolipids are associated with their amphiphilic action on cellular membranes and the activation of mitochondrial and ER stress pathways, without involving classical apoptosis pathways. Thus, sophorolipids emerge as promising natural antiproliferative agents, with multiple intracellular targets, and support further investigations that explore their efficacy in vivo models.

## Supplementary Information

Below is the link to the electronic supplementary material.

Supplementary Material 1

Supplementary Material 2

Supplementary Material 3

Supplementary Material 4

Supplementary Material 5
