# Supplementary material for: Investigation of the molecular mechanisms of action of sophorolipids in MCF-7 cells: therapeutic potential against breast cancer
Source: 3 Biotech. 2026 Oct 23;16(11):471. doi: 10.1007/s13205-026-05096-0 (PMC13694592; doi:10.1007/s13205-026-05096-0)
Supplement: Supplementary file 1 [file 13205_2026_5096_MOESM5_ESM.docx]

**Table S1** Forward and reverse primer sequences of the target genes used in RT–PCR.

| ***Gene*** | **Forward** | **Reverse** |
| --- | --- | --- |
| *ACTB* | 5’ GAC GAC ATG GAG AAA ATC TG 3’ | 5’ ATG ATC TGG GTC ATC TTC TC 3’ |
| *BBC3* | 5' GTA AGA TAC TGT ATA TGC GCT G 3' | 5' TTT TCC ACT GTT CCA ATC TG 3' |
| *BCL2* | 5' GAT TGT GGC CTT CTT TGA G 3' | 5' GTT CCA CAA AGG CAT CC 3' |
| *BECN1* | 5’ CAG TAT CAG AGA GAA TAC AGT G 3’ | 5’ TGG AAG GTT GCA TTA AAG AC 3’ |
| *CDKN1A* | 5' CAG CAT GAC AGA TTT CTA CC 5' | 5' CAG GGT ATG TAC ATG AGG AG 3’ |
| *C-MYC* | 5' TGA GGA GGA ACA AGA AGA TG 3' | 5' ATC CAG ACT CTG ACC TTT TG 3' |
| *ERN1* | 5' ACG GAC GTC AAG TTT GAT CC 3' | 5' TTG GTA GAC GCA GAC AGT GG 3' |
| *GAPDH* | 5' GAA GGT GAA GGT CGG AGT C 3' | 5' GGA AGA TGG TGA TGG GAT TT 3' |
| *GADD45A* | 5' TCA GCG CAC GAA GAC TGT C 3' | 5' CCA GCA GGC ACA ACA CCA C 3' |
| *NFKB1* | 5' GCA GCT CTT CTC AAA GCA GCA 3’ | 5' GCT CAA AGT TCT CCA CCA GGG 3' |
| *MLKL* | 5’ GTG AAG AAT GTG AAG ACT GG 3’ | 5’ AAG ATT TTC ATC CAC AGA GGG 3’ |
| *RPL0* | 5’AGATGCAGCAGATCCGCAT 3’ | 5’ GTGGTGATACCTAAAGCCTG 3’ |

**Table S2 -** Analysis of mRNA expression of target genes using RT-qPCR.

| Genes | Fold Change | Log_2_FC |
| --- | --- | --- |
| ***ACTB*** | 0,192 ± 0,068 * | -2,883 ± 1,414 |
| ***BBC3*** | 3,832 ± 1,259 * | 2,541 ± 1,343 |
| ***BCL2*** | 1,380 ± 0,309 | 0,446 ± 0,326 |
| ***BECN1*** | 1,339 ± 0,296 | 0,402 ± 0,326 |
| ***C-myc*** | 3,071 ± 0,145 * | 1,618 ± 0,676 |
| ***CDKN1A*** | 22,73 ± 3,359 * | 4,496 ± 0,216 |
| ***ERN1*** | 10,57 ± 0,000 * | 3,402 ± 0,000 |
| ***GADD45A*** | 8,646 ± 0,556 * | 3,110 ± 0,092 |
| ***GAPDH*** | 0,377 ± 0,144 * | -1,459 ± 0,564 |
| ***MLKL*** | 0,242 ± 0,124 * | -2,166 ± 0,700 |
| ***NF-Kβ*** | 1,346 ± 0,215 | 0,419 ± 0,321 |

Relative expression of mRNAs in MCF-7 cells treated with sophorolipids. The values are presented as mean ± standard deviation of three independent experiments. The analysis was performed considering significant differences when *fold change* ≤ 0.5 (repression) or ≥ 2 (overexpression), with a significance level of 5% (p < 0.05). The symbol (*) indicates a statistically significant difference from the control
